# Nitric Oxide Deficiency Accelerates Chlorophyll Breakdown and Stability Loss of Thylakoid Membranes during Dark-Induced Leaf Senescence in Arabidopsis

**DOI:** 10.1371/journal.pone.0056345

**Published:** 2013-02-13

**Authors:** Fang Liu, Fang-Qing Guo

**Affiliations:** The National Key Laboratory of Plant Molecular Genetics and National Center for Plant Gene Research (Shanghai), Institute of Plant Physiology & Ecology, Shanghai Institutes for Biological Sciences, Chinese Academy of Sciences, Shanghai, People's Republic of China; RIKEN Biomass Engineering Program, Japan

## Abstract

Nitric oxide (NO) has been known to preserve the level of chlorophyll (Chl) during leaf senescence. However, the mechanism by which NO regulates Chl breakdown remains unknown. Here we report that NO negatively regulates the activities of Chl catabolic enzymes during dark-induced leaf senescence. The transcriptional levels of the major enzyme genes involving Chl breakdown pathway except for *RED CHL CATABOLITE REDUCTASE* (*RCCR*) were dramatically up-regulated during dark-induced Chl degradation in the leaves of Arabidopsis NO-deficient mutant *nos1/noa1* that exhibited an early-senescence phenotype. The activity of pheide *a* oxygenase (PAO) was higher in the dark-induced senescent leaves of *nos1/noa1* compared with wild type. Furthermore, the knockout of *PAO* in *nos1/noa1* background led to pheide *a* accumulation in the double mutant *pao1 nos1/noa1*, which retained the level of Chl during dark-induced leaf senescence. The accumulated pheide *a* in darkened leaves of *pao1 nos1/noa1* was likely to inhibit the senescence-activated transcriptional levels of Chl catabolic genes as a feed-back inhibitory effect. We also found that NO deficiency led to decrease in the stability of photosynthetic complexes in thylakoid membranes. Importantly, the accumulation of pheide *a* caused by *PAO* mutations in combination with NO deficiency had a synergistic effect on the stability loss of thylakoid membrane complexes in the double mutant *pao1 nos1/noa1* during dark-induced leaf senescence. Taken together, our findings have demonstrated that NO is a novel negative regulator of Chl catabolic pathway and positively functions in maintaining the stability of thylakoid membranes during leaf senescence.

## Introduction

Chlorophyll (Chl) molecules play a central role in the initial and indispensable processes of photosynthesis, such as harvesting light energy and driving electron transfer. However, like most porphyrins, Chl is also a dangerous molecule and potentially hazardous to plant cells in situations where the photosynthetic apparatus is overexcited and absorbed energy is transferred from chl to oxygen, resulting in the production of reactive oxygen species (ROS) [1], [2]. Indeed, plant cells need a process to inactivate this hazard efficiently through a Chl catabolism pathway. As a dramatically visualized sign of leaf senescence and fruit ripening, loss of green color is resulted from Chl breakdown combined with carotenoid retention or anthocyanin accumulation [2], [3]. Thus, the degradation of Chl is a prerequisite to detoxify the potentially phototoxic pigments in order to remobilize the nitrogen pools of the apoproteins from Chl-binding proteins in chloroplasts during leaf senescence [2]–[5].

In recent years, important progresses have been made in better understanding the pathway of Chl catabolism in higher plants. In brief, the initial reaction during the Chl breakdown pathway is the removal of the phytol residue and the central Mg by chlorophyllase and metal chelating substance, respectively. In 1999, Chlorophyllase genes, termed *CLHs*, were cloned in Arabidopsis leaves [6]. However, recent studies questioned the involvement of *CLHs* in Chl breakdown *in vivo* during leaf senescence [7], [8] even though the two CLHs present in Arabidopsis exhibited chlorophyllase activity *in vitro* [6]. In 2009, pheophytinase (PPH) was identified to function in porphyrin-phytol hydrolysis involved in senescence-related chlorophyll breakdown *in vivo* [9]. In the next-step reactions, the resulting pheophorbide (pheide) *a* is converted into a primary fluorescent chlorophyll catabolite (pFCC), which requires two enzymes including pheide *a* oxygenase (PAO) and red chl catabolite reductase (RCCR) [10]–[13]. *PAO* is identical to *ACCELERATED CELL DEATH* (*ACD1*) and encodes a chloroplast envelop-bound Rieske-type iron-sulfur oxygenase [12]. Unlike PAO, RCCR is a soluble protein targeted to chloroplasts [11]. PAO catalyzes the cleavage of the porphyrin ring, resulting in the red chlorophyll catabolite, which is further reduced in to pFCC by RCCR. The resulting primary fluorescent catabolite pFCCs are exported from the plastid and after further modification they are imported into their final destination, the vacuole by a primary active transport system [14], [15].

Recently, the genetic lesion identification of stay-green mutants has provided an in-depth understanding of chlorophyll degradation pathway. *NON-YELLOW COLORING1* (*NYC1*) and *NYC1-LIKE* (*NOL*) were cloned based on the genetic analysis of the stay-green mutants *nyc1* and *nol*, which selectively retained photosystem II (PSII) light-harvesting complex subunits and exhibited high concentrations of chlorophyll *b* [16], [17]. *NYC1* and *NOL*, encoding two subunits of chlorophyll *b* reductase, catalyze the first half of chlorophyll *b* to chlorophyll *a* reduction [17]. Besides *nyc1*/*nol*, the mutation of the *PAO* gene caused a stay-green phenotype during dark-induced leaf senescence. It was reported that the *pao* mutant plants showed a light-dependent lesion mimic phenotype due to the accumulation of phototoxic pheide *a* [12], [18], [19]. Recent studies on plant senescence research have been focusing on the third type of stay-green mutants, which are defective in a stay-green gene, termed *senescence-induced degradation* (*SID*) in the Bf993 mutant of *Festuca pratensis* [20]–[22]. The orthologous genes of *SID*, now designated *SGR* (*STAY-GREEN*), have been identified in a variety of plant species such as Arabidopsis [23], rice [24]–[26], pea [26], [27], bell pepper [28], [29], and tomato [28]. Interestingly, SGR directly interacts with a subset of the proteins in the light harvesting chlorophyll a/b-protein complex II (LHCPII), implying that SGR may be involved in destabilizing pigment-protein complexes as a prerequisite for chlorophyll degrading enzymes to access their substrate during leaf senescence [2], [25], [30].

Although tremendous progress has been made in the elucidation of the mechanism through which chlorophyll is degraded in the last decade, our current knowledge of the regulatory networks of chlorophyll breakdown during senescence remains limited [2], [3], [30]. Given that plant hormones regulate senescence processes marked as Chl breakdown, it is not unexpected that Chl catabolic gene expression is under hormonal control. The senescence-promoting hormones ethylene and MeJA stimulate CLH activity [31]. The treatment of MeJA activates the expression of *AtCLH1* in Arabidopsis [6], and in *Citrus*, *CLH1* is highly up-regulated by ethylene [32]. As a key player of Chl breakdown pathway, *PAO* is highly expressed in senescent tissues, but at low levels in presenescent leaves [18]. Like *AtCLH1* [33], the expression of *PAO* is also responsive to wounding [34]. Generally, it remains to be shown which hormonal regulators or messenger molecules are critical for the regulation of Chl degradation pathway.

NO has been noted as an antisenescence signal in that NO treatments extend the postharvest life of fruits and vegetables [35]. During storage, NO application delays yellowing and retards the onset of chlorophyll degradation in broccoli (*Brassica oleracea*) florets [36], [37]. NO preserves the level of Chl in potato leaves infected by *Phytophthora infestans* [38] and counteracts leaf senescence induced by MeJA [39]. In pea (*Pisum sativum*), NO levels are significantly reduced during the natural senescence [40] and negatively correlate with ethylene levels [35], [41]. NO treatments can protect against senescence-dependent chlorophyll degradation in soybean (*Glycine max*) cotyledons [42]. On the other hand, leaf senescence occurs more rapidly in NO-deficient mutants compared with wild type plants [43], [44].

In this study, we have explored the physiological effects of the reduced endogenous NO levels on Chl degradation during dark-induced leaf senescence using the NO-deficient mutant *nos1/noa1* [45] in combination with the expression analysis of Chl catabolic genes and the enzymatic activity examination of PAO. Although the role of NOS1/NOA1 in NO biosynthesis remains controversial [45]–[49], it has been demonstrated that the mutation of *NOS1/NOA1* causes a significant reduction in NO production in *Arabidopsis* [45], [50]–[54] and *Nicotiana benthamiana* [55]. Recent data suggested that NOS1/NOA1 is a functional cGTPase essential for chloroplast function [56], [57] and its role in endogenous NO accumulation needs to be further explored [49], [58], [59]. Interestingly, mutant NOS1/NOA1 lacking the C-terminal domain, although retaining GTPase activity, failed to complement the mutant *nos1/noa1*, suggesting that this GTPase activity of NOS1/NOA1 is not sufficient to recover the NO deficiency-related phenotypes of the *nos1/noa1* plants [49]. In this study, we observed that the rapid loss of Chl occurring in the NO-deficient mutant *nos1/noa1*in darkness is attributable to the up-regulated expression of the major genes involved in Chl degradation pathway. Here, we also show that the knockout of *PAO* in the *nos1/noa1* mutant retains levels of Chl during dark-induced leaf senescence, suggesting that the *PAO* mutation-caused accumulation of Chl catabolite pheide *a* is most likely to inhibit the rapid degradation of Chl resulted from NO deficiency in the *nos1/noa1* mutant. Our findings support the hypothesis that NO functions as an anti-senescence messenger molecule through repressing the transcriptional activation of the major Chl catabolic genes during leaf senescence.

## Results

### NO represses the transcriptional activation of Chl catabolic pathway genes during dark-induced leaf senescence

**Figure 1:**
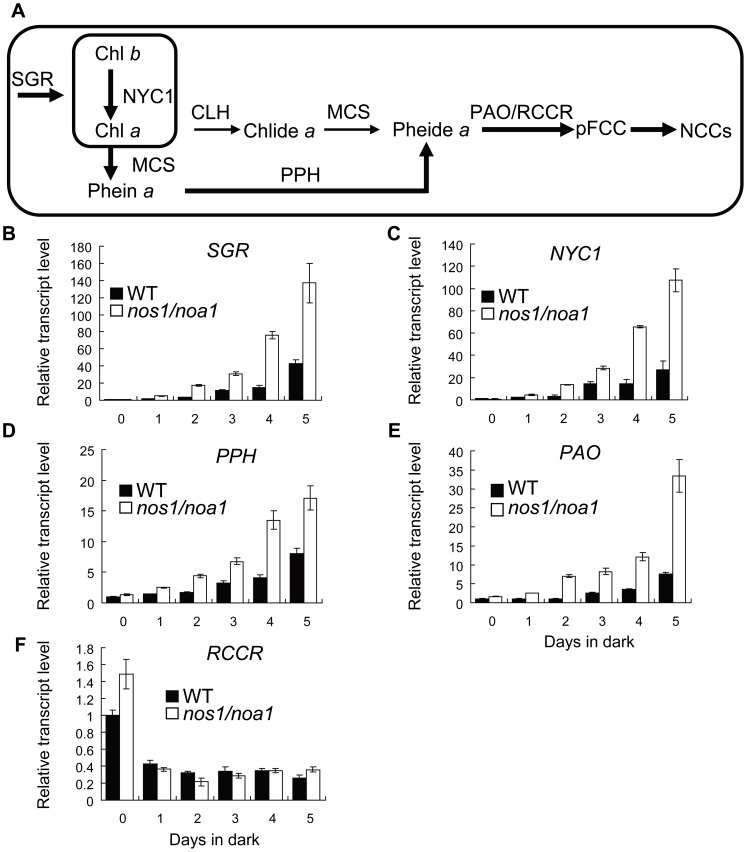
Transcript levels of chlorophyll breakdown pathway genes in wild type and *nos1/noa1* mutant leaves during dark-induced senescence. (A) A suggestive model for chlorophyll breakdown pathway with integrating the recent findings in Arabidopsis. Thickness of arrows reflects relative activities of respective enzymes (Hörtensteiner, 2009). (B–F) qRT-PCR analysis of mRNA abundance of enzyme genes (*SGR*, *NYC1*, *PPH*, *PAO* and *RCCR*) involved in chlorophyll degradation in wild type and *nos1/noa1* mutant leaves during dark-induced senescence. *ACTIN2* was used as the internal standard. Error bars indicate standard deviations of three technical replicates, and the results were consistent in three biological replicates.

Given that the depletion of endogenous NO leads to an early leaf senescence phenotype in the mutant plants [43], [44], we investigated whether NO plays a role in the regulation of Chl breakdown pathway during leaf senescence using the NO-deficient mutant *nos1/noa1*. The quantitative reverse transcription PCR technique was used to examine the relative levels of the major genes involving Chl breakdown pathway in the wild type and *nos1/noa1* mutant leaves during dark-induced leaf senescence. Upon dark treatment for 5 d, the transcription levels of *SGR*, *NYC1*, *PPH* and *PAO* were dramatically up-regulated in the *nos1/noa1* mutant compared with that in wild type (Fig. 1B–E). The strongest increase (3.4-fold) was detected for *PAO*, and the increase range for the rest of the genes was from 1.1 to 2.9-fold when leaves were dark-treated to induce senescence. In contrast to the four genes mentioned above, the mRNA level of *RCCR* was down-regulated at day one and was kept at similar levels in both the wild type and *nos1/noa1* mutant leaves through to day 5 in darkness, which is consistent with the previous results that *RCCR* expression is constitutively active throughout the process of leaf senescence [60], [61]. Interestingly, we found that sodium nitroprusside (SNP) treatment (250 µM) dramatically inhibited the transcriptional activation of Chl breakdown pathway genes, including *SGR*, *NYC1*, *PPH* and *PAO* in the *nos1/noa1* mutant compared with the mutant leaves under control treatment (Fig. S1). On the other hand, we have also performed the NO depletion experiments to further validate our hypothesis using the widely-used NO specific scavenger, cPTIO. We found that upon dark treatment for 4 d, the transcription levels of SGR, NYC1, PPH and PAO were more dramatically up-regulated in the darkened leaves of wild type treated with 500 µM 2-(4-carboxyphenyl)-4,4,5,5-tetramethylimidazoline-1-oxyl-3 oxide (cPTIO) compared with the leaves of wild type under control condition, which mimics the expression patterns of Chl catabolic genes in the NO-deficient mutant *nos1/noa1* during dark-induced leaf senescence (Fig. S2). In addition, it should be mentioned that no significant difference was observed in the transcript levels of *SGR*, *NYC1*, *PPH* and *PAO* between the wild type and *nos1/noa1* mutant at the beginning of dark-treatment. These results indicate that NO represses the transcriptional activation of the major genes involving Chl catabolic pathway during dark-induced leaf senescence.

### NO inhibits PAO activity during dark-induced leaf senescence

**Figure 2:**
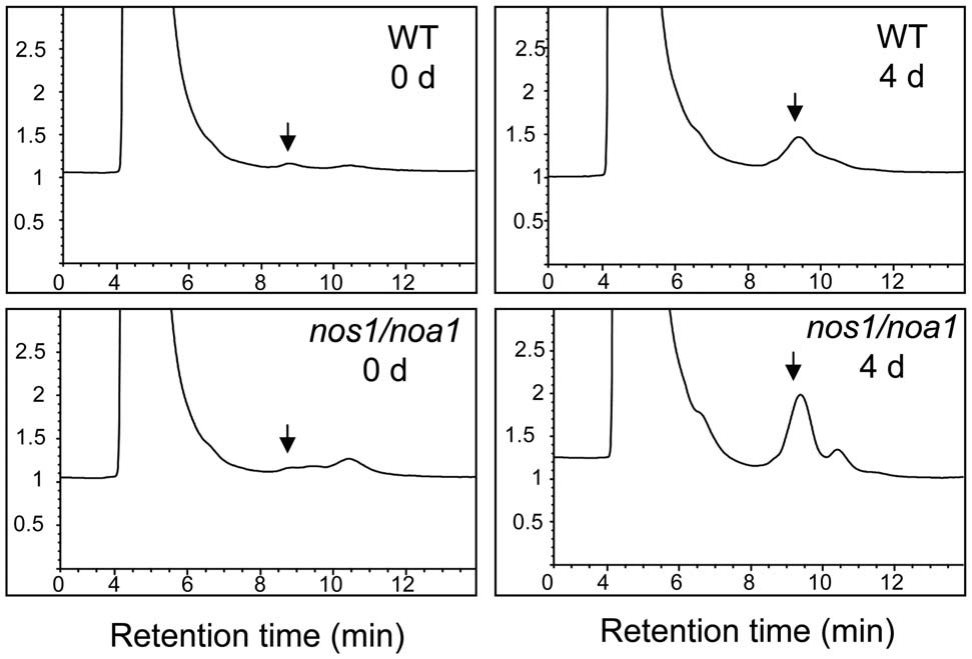
Activities of PAO in wild type and *nos1/noa1* leaves during dark-induced leaf senescence. HPLC analysis of pFCC-1 generated by PAO-RCCR coupled assay with crude extracts of PAO and RCCR from wild type and *nos1/noa1* leaves during a 4-d dark treatment. pFCC-1 was eluted after 9 min, as indicated by arrows. The experiment was repeated once with qualitatively identical results.

In Arabidopsis, the activity of PAO is enhanced in senescent leaves [12]. To verify the effect of NO on the activity of PAO during dark-induced leaf senescence, we assessed in vivo PAO activity using PAO-RCCR coupled assay with crude extracts of PAO and RCCR from the wild type and *nos1/noa1* mutant leaves, respectively, during a 4-d dark treatment. The results showed that the activity of PAO in the *nos1/noa1* mutant was significantly higher than that in wild type at day 4 in darkness, although the activities of PAO in both genotypes increased upon dark treatment (Fig. 2 and Table S1). These data further support that NO acts as a negative regulator in modulating chlorophyll breakdown during leaf senescence.

### *PAO* mutations delay the rapid Chl degradation in the *nos1/noa1* mutant

**Figure 3:**
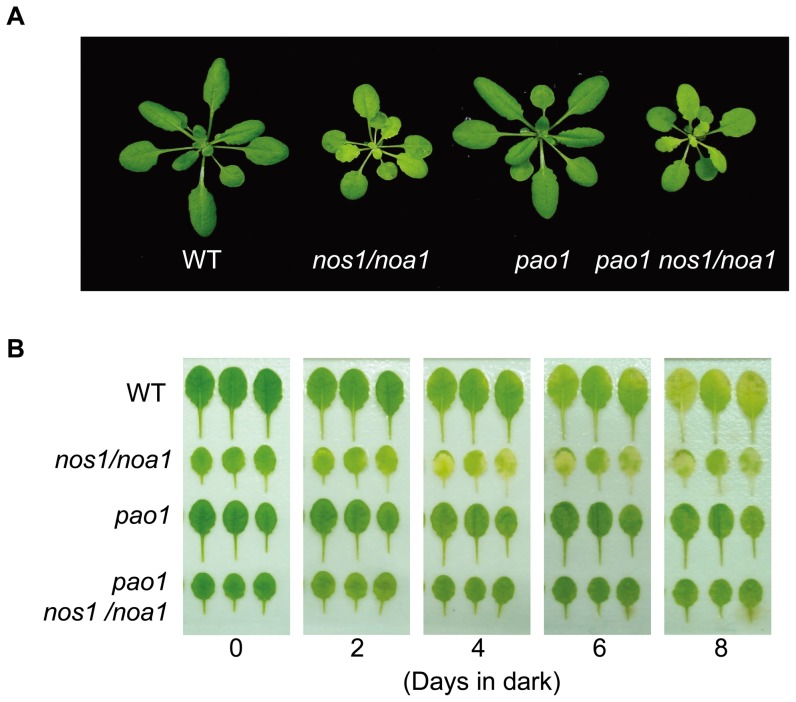
Dark-induced leaf senescence phenotypes of wild type, *nos1/noa1*, *pao1* and the double mutant *pao1 nos1/noa1*. (A) 3-week-old seedling phenotypes of wild type, *nos1/noa1*, *pao1* and the double mutant *pao1 nos1/noa1*. (B) Senescing phenotypes of the detached leaves in dark. The fully-expanded leaves were detached from the plants of the different genotypes shown in (A).

**Figure 4:**
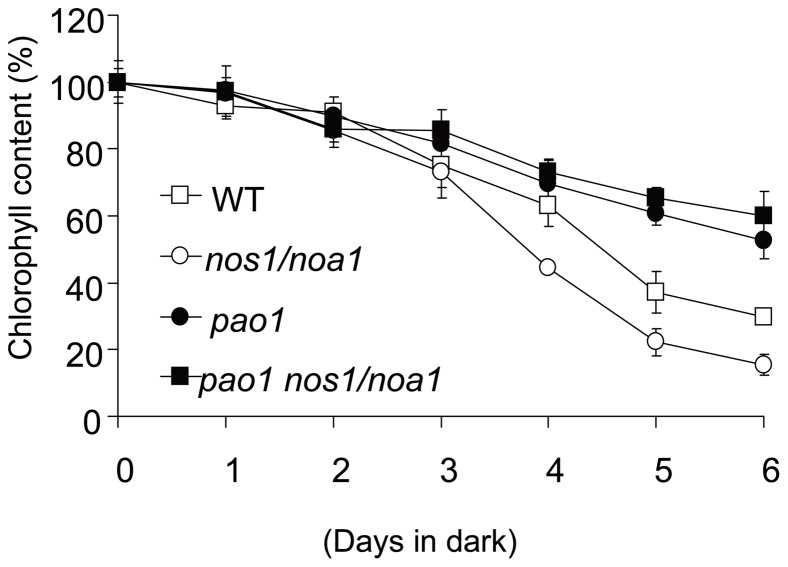
Analysis of chlorophyll contents during dark-induced leaf senescence. Chlorophyll contents were measured in the detached leaves of the indicated genotypes during a 6-d-dark treatment. Data are means of three replicates. Error bars indicate SD.

Chl degradation occurs rapidly in the darkened leaves of *nos1/noa1* mutant due to endogenous NO deficiency [43]. It is also well known that in *pao* mutants, Chl is largely retained during senescence [12], [18], which supports the hypothesis that a blockage at the level of PAO inhibits Chl breakdown. To address the question whether the rapid degradation of Chl in the *nos1/noa1* mutant during senescence can be inhibited by the accumulation of Pheide *a* caused by *PAO* mutations, we generated the double mutant by crossing the *nos1/noa1* mutant to a loss-of-function mutant *pao1*. Interestingly, the double mutant plants showed a smaller size with pale-green leaves just like *nos1/noa1* mutant plants (Fig. 3A). When compared with the leaves of the *nos1/noa1* mutant, the leaves of the identified double mutant *pao1 nos1/noa1* were found to retain the Chl levels as well as the *pao1* single mutant throughout to day 8 in darkness (Fig. 3B). For instance, the levels of Chl contents in the double mutant and *pao1* were only reduced to around 60% of the initial levels at day 6 upon dark-treatment, whereas the levels of Chl contents in the wild type and *nos1/noa1* mutant were severely reduced to around 30% and 20%, respectively (Fig. 4A). Hence, the dark-induced rapid degradation of Chl in the *nos1/noa1* mutant was reversed by *PAO* mutations. These data provided evidence that the accumulation of Chl catabolite Pheide *a* is most likely to inhibit the rapid Chl breakdown caused by NO-deficiency as exhibited in the *nos1/noa1* mutant.

**Figure 5:**
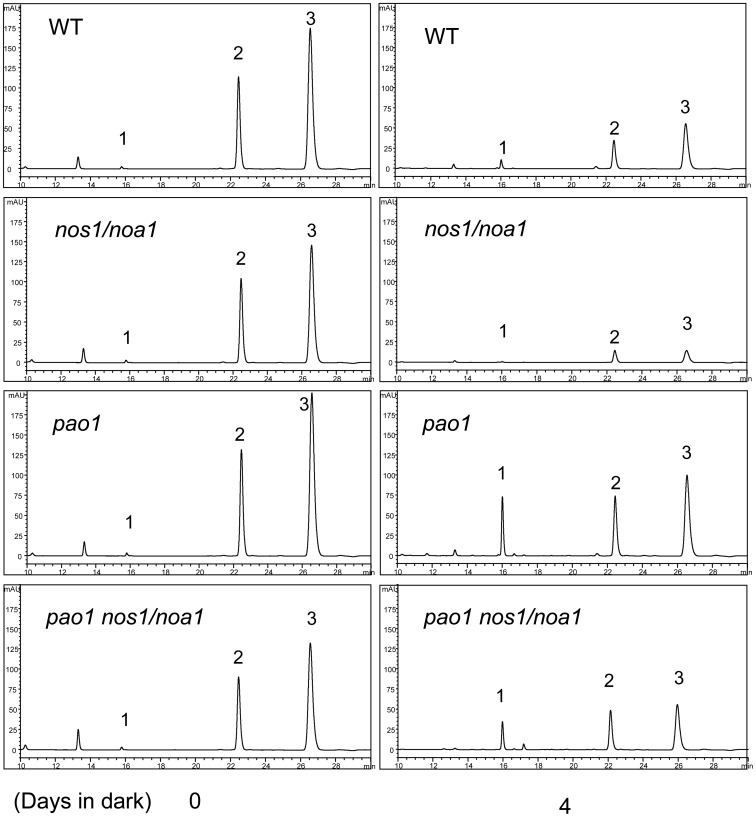
Green pigment changes in the leaves of wild type, *nos1/noa1*, *pao1* and *pao1 nos1/noa1* during dark-induced leaf senescence by HPLC analysis. Representative data of green pigment changes in the leaves of the indicated genotypes after a 4-d-dark incubation. Numerals 1 to 3 indicate Pheide *a*, Chl *b*, and Chl *a*, respectively. The experiment was repeated twice.

**Figure 6:**
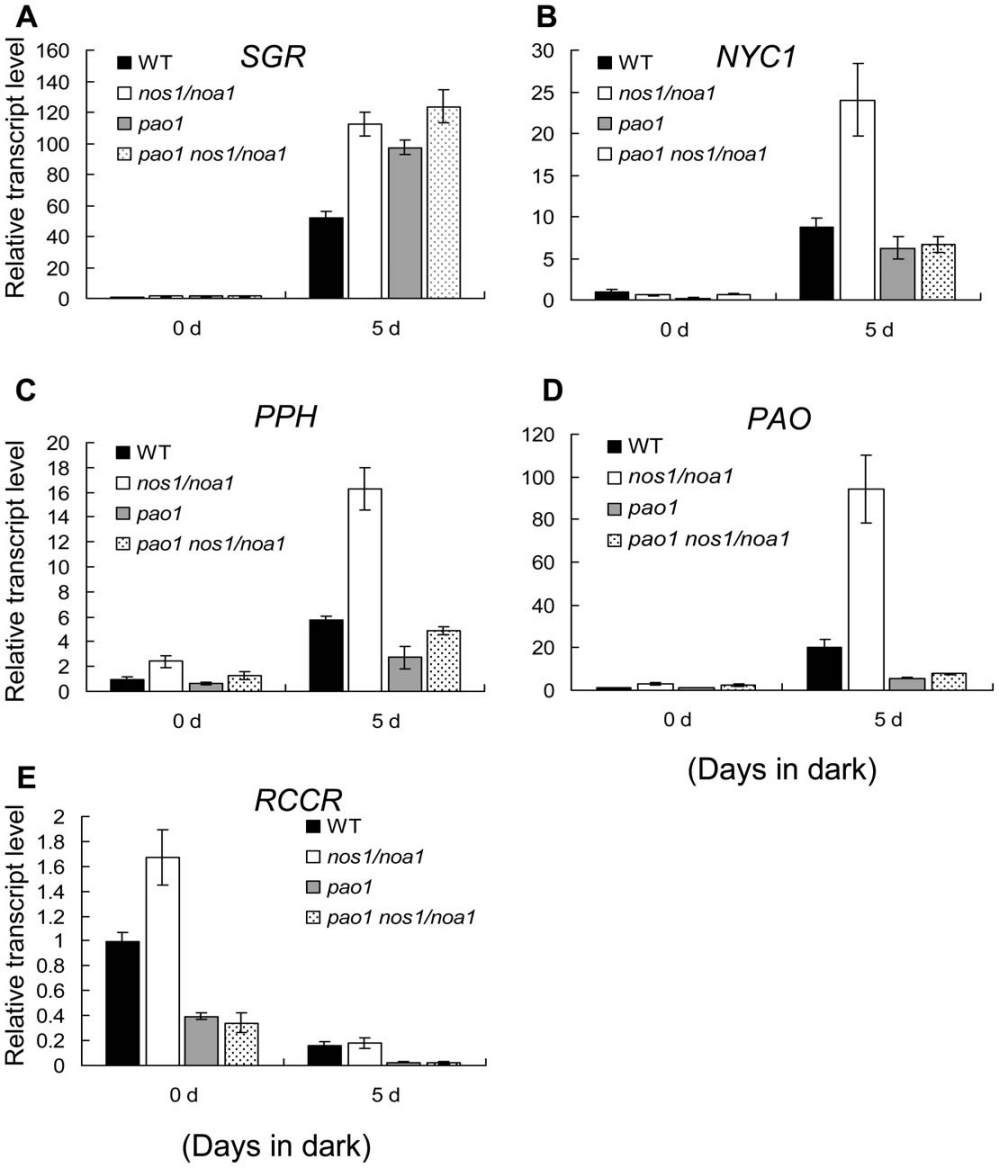
The effects of accumulated Pheide *a* on the transcript levels of chlorophyll breakdown pathway genes during dark-induced senescence. (A–E) qRT-PCR analysis of mRNA abundance of chlorophyll catabolic genes (*SGR*, *NYC1*, *PPH*, *PAO* and *RCCR*) in wild type and the indicated mutant leaves during dark-induced senescence. *ACTIN2* was used as the internal standard. Error bars indicate standard deviations of three technical replicates, and the results were consistent in three biological replicates.

To further investigate whether the endogenous Pheide *a* accumulation in the double mutant *pao1 nos1/noa1* affects the rapid up-regulation of Chl catabolic pathway genes, we verified the expression patterns of *SGR*, *NYC1*, *PPH*, *PAO* and *RCCR* in the mutants and wild type leaves after dark-treatment by qRT-PCR. Interestingly, we found that the senescence-induced up-regulation of *NYC1*, *PPH* and *PAO* in the double mutant *pao1 nos1/noa1* leaves, in which the Pheide *a* accumulation was validated using HPLC (Fig. 5), was dramatically inhibited in comparison with that in the *nos1/noa1* mutant and wild type leaves (Fig. 6B–D). It should be noted that the up-regulated expression of *SGR* was not significantly affected in the double mutant in reference to the expression level of *SGR* in the *nos1/noa1* mutant (Fig. 6A), indicating that the blockage of Chl degradation pathway at PAO has little feed-back inhibitory effect on *SGR* expression. These results suggest that the accumulation of Pheide *a* caused by *PAO* mutations might act as feed-back signals to repress the senescence-activated expression of the major genes involving Chl catabolic pathway.

### NO deficiency impairs the stability of photosynthetic complexes during dark-induced leaf senescence

**Figure 7:**
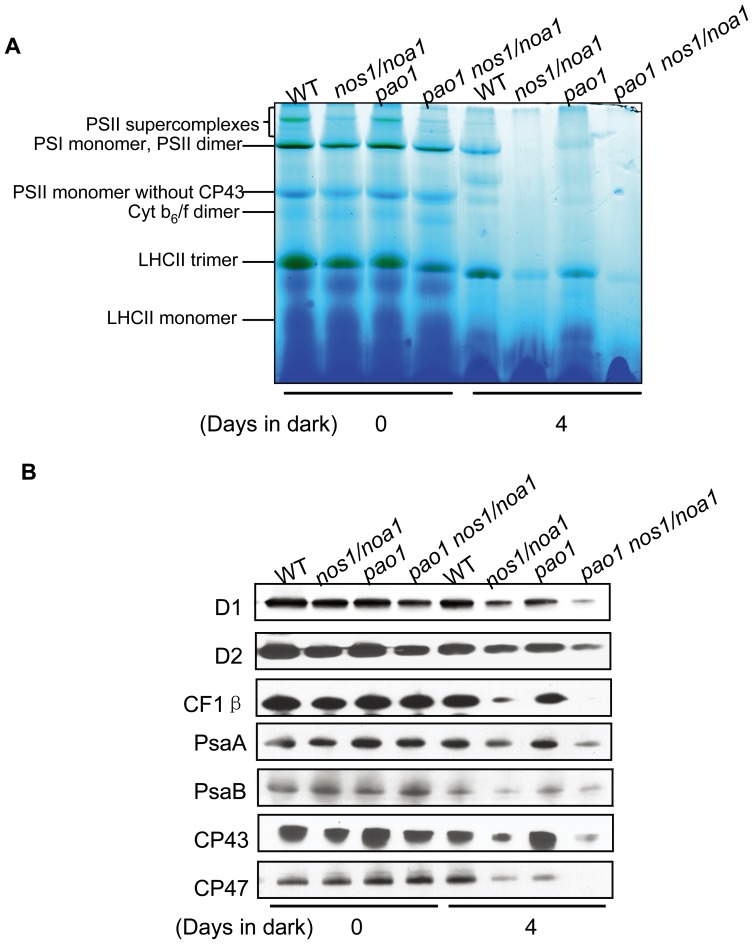
Abundance analysis of thylakoid membrane protein complexes from leaves incubated in dark. (A) Blue native-PAGE analysis of thylakoid membrane protein complexes from the detached leaves of wild type and the indicated mutants after a 4-d-dark treatment. (B) Western blotting analysis of thylakoid membrane protein degradation from the detached leaves of wild type and the indicated mutants after a 4-d-dark treatment.

Given that NO deficiency accelerates Chl breakdown during senescence, it is attempted to test whether NO modulates degradation of photosynthetic complexes upon dark-treatment. Thylakoid membranes were isolated from the detached leaves incubated in darkness for 4 d and solubilized mildly with detergent (dodecyl β-D-maltoside). The resulting thylakoid membrane protein complexes were separated by Blue-Native gel electrophoresis (BN-PAGE) under native conditions in the presence of Serva Blue G. As shown in Fig. 7A, the typical thylakoid membrane protein complexes of the wild type include the higher molecular weight complexes corresponding to PSII supercomplexes, the PSI monomer and PSII dimer, PSII core monomer, PSII monomer without the CP43 subunit, Cyt b_6_/f dimer, LHCII trimer and LHCII monomer. Notably, all of the thylakoid membrane protein complexes were extremely less abundant in the *nos1/noa1* mutant as well as in the double mutant *pao1 nos1/noa1* after a 4-d dark treatment in comparison with that in wild type (Fig. 7A), indicating that NO deficiency destabilizes all of the thylakoid membrane protein complexes during dark-induced leaf senescence, and the Chl retention caused by the mutations of *PAO* in the double mutant does not improve the stability of the indicated complexes. Moreover, all of the thylakoid membrane protein complexes were less abundant in the wild type leaves treated with cPTIO after a 4-d dark treatment in comparison with that in wild type under control conditions (Fig. S3), indicating that NO deficiency induced by cPTIO treatments destabilizes all of the thylakoid membrane protein complexes during dark-induced leaf senescence. Interestingly, the abundance of the thylakoid membrane protein complexes in the *pao* mutant was significantly higher than that in the double mutant *pao1 nos1/noa1* after a 4-d dark treatment, implying that NO deficiency and the accumulation of Pheide *a* may have synergistic effects on the stability loss of the indicated complexes (Fig. 7A). In addition, we observed that under normal growth conditions, the abundance of the indicated complexes in both the *nos1/noa1* mutant and the *pao1 nos1/noa1* double mutant was slightly reduced compared with the wild type, whereas the *pao1* mutant exhibited a similar abundance level to the wild type (Fig. 7A), supporting that NO is a positive regulator in stabilizing thylakoid membranes under both normal and stress conditions.

To further explore the effects of NO on the stability of thylakoid membrane proteins, western blotting was performed to examine the abundance of photosynthetic proteins in wild type and the indicated mutants upon dark treatment as shown in Fig. 7B. As revealed by western blotting data, substantial amounts of photosynthetic proteins were lost in both the *nos1/noa1* mutant and the *pao1 nos1/noa1* double mutant 4 d after dark treatment with respect to the wild type, but the abundance levels of the indicated photosynthetic proteins were quite similar between the wild type and the *pao1* mutant (Fig. 7B).

**Figure 8:**
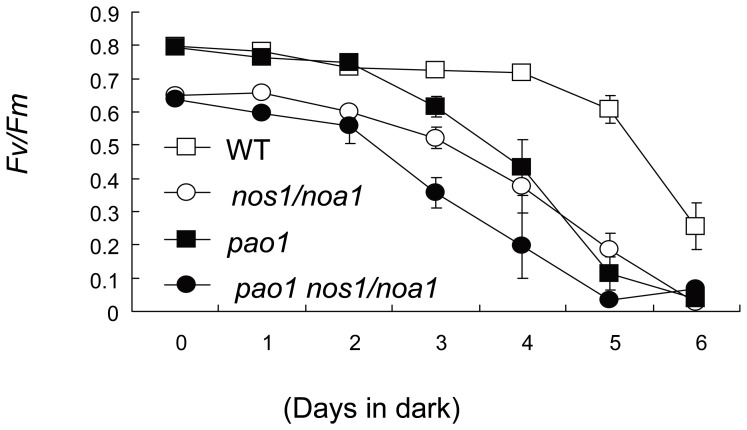
Analysis of PSII photochemical efficiency (Fv/Fm) of detached leaves during dark-induced senescence. The fifth leaves were excised from wild type and the indicated mutant plants (21-d-old). Error bars indicate SE (n = 5).

Given that the significant changes in protein abundance of PSII subunits as observed in the indicated mutant leaves upon dark treatment, the photosynthetic Chl fluorescence parameter *Fv/Fm* was measured in order to test the maximal quantum yield of PSII. Interestingly, a typical *Fv/Fm* value (0.8) for healthy plants was measured in wild-type leaves, whereas the *Fv/Fm* ratio in both the *nos1/noa1* mutant and the *pao1 nos1/noa1* double mutant leaves was around 0.65, indicating a rather severe defect in PSII (Fig. 8). During the dark treatment, the *Fv/Fm* values of both the *nos1/noa1* mutant and the *pao1 nos1/noa1* double mutant leaves were found to be 48% and 73% less compared with the wild type respectively, when leaves were darkened for 4 d (Fig. 8). Consistent with the results obtained in BN-PAGE (Fig. 7A) and western blotting (Fig. 7B), it is noteworthy that the *Fv/Fm* ratio of the *pao1* mutant leaves was less than 40% reduced in comparison with the wild type leaves after 4-d dark treatment. The *Fv/Fm* values of all the mutant leaves decreased progressively during the following days. By contrast, the *Fv/Fm* in the wild type leaves only slightly decreased from 0.8 to 0.69 through 5 d in darkness and dropped at day 6 due to a large decrease in PSII quantum efficiency (Fig. 8). In the darkened leaves of the indicated mutants as shown in Fig. 8, dark-induced PSII damages were more pronounced, and PSII damaging proceeded at a faster rate relative to wild-type leaves.

**Figure 9:**
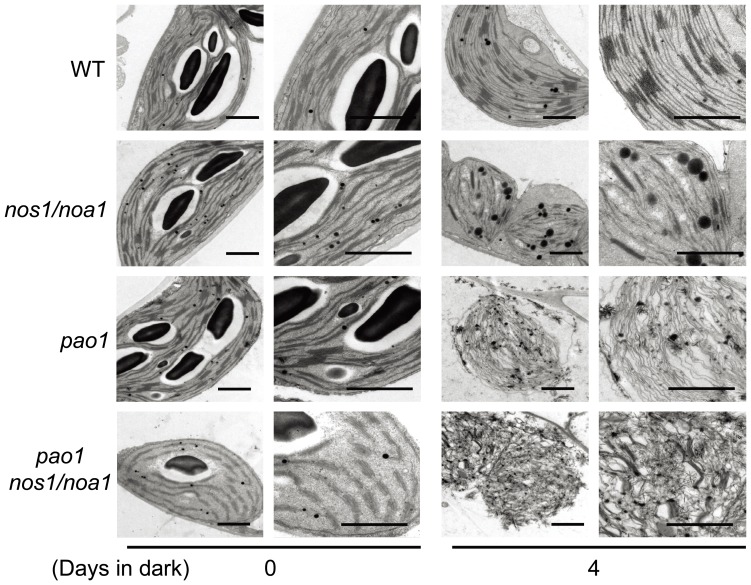
Chloroplast ultrastructures of detached leaves during dark-induced senescence. Cross-sectional analysis of thylakoid membranes in chloroplasts from the fully-expanded leaves detached from wild type, *nos1/noa1*, *pao1* and the double mutant *pao1 nos1/noa1* plants after 0 d and 4 d of dark-treatment by transmission electron microscopy (TEM). Low magnification overview (left) and close-up images (right) of the chloroplast ultrastructure were shown respectively at day 0 and day 4. Bars = 1 µm.

To further explore the effect of NO deficiency on the stability maintenance of thylakoid membranes, we conducted examinations of chloroplast ultrastructural alterations by transmission electron microscopy (TEM). The thylakoid membranes in the chloroplasts of the mutant *nos1/noa1* appeared distorted and began to decompose after 4 d dark-treatment, whereas wild type thylakoid systems, including grana and stroma membranes, retained their initial configurations (Fig. 9). In agreement with the findings by BN-PAGE and western blotting, detailed examinations by TEM revealed that the ultrastructures of chloroplasts in the double mutant *pao1 nos1/noa1* were most severely disrupted and thylakoid membranes were almost totally decomposed with respect to that in wild type, *pao1* and *nos1/noa1* mutants upon a 4-d dark treatment (Fig. 9). Thus, the findings by TEM examinations further support that NO deficiency and the accumulation of Pheide *a* have synergistic effects on the stability loss of thylakoid membranes upon dark-treatment. Taken together, these data support the notion that NO deficiency affects the maximal quantum yield of PSII through modulating the stability of thylakoid membranes during dark-induced leaf senescence.

## Discussion

Accumulated studies pointed to the critical roles of plant hormones in triggering, accelerating or even reversing the overall process of senescence by activating specific genes [62]–[66]. It appears that plants may integrate these different hormonal signaling factors through endogenous signaling networks in order to decide whether the senescence process should be executed or not. Two classical hormones ethylene and cytokinin have been recognized to influence leaf senescence through acting as a senescence-promoting factor and as a senescence-delaying factor, respectively in higher plants [67], [68]. Although it has been noted that NO plays a role in senescence by retarding the onset of chlorophyll degradation in different plant species [36]–[39], [42], the mechanisms through which NO modulates Chl degradation during senescence remain elusive.

In this study, the NO-deficient mutant *nos1/noa1* was employed to address the effect of NO on Chl breakdown during leaf senescence since the dark-induced leaf senescence occurs more rapidly in the mutant compared with wild type plants [43]. Considering that leaf senescence occurs sequentially in individual leaves within the rosette rather than in the whole plant in Arabidopsis [69], dark-induced senescence of detached leaves was employed in this study. Our present results demonstrate that the expression of the genes encoding enzymes involved in Chl catabolism is severely repressed, except for *RCCR* during dark-induced leaf senescence. In our senescence induction system, *RCCR* is not an inducible gene upon dark treatment, whereas its transcript level is slightly reduced during senescence. Our data further confirm that RCCR seems to be constitutively active and its activity is rather constant during all phases of leaf development [5], [18].

The hypothesis underlying the findings presented in this report is that endogenous NO deficiency caused by *NOS1/NOA1* mutations leads to loss of signal that represses the transcriptional activation of Chl catabolic genes during dark-induced leaf senescence. To our knowledge, endogenous NO, acting as a negative messenger molecule in regulating Chl breakdown at transcriptional level during leaf senescence, has not been previously reported. Previous reports suggest that endogenous NO levels negatively correlate with senescence progression in many different plant species. Nitric oxide (NO) has been characterized as an anti-senescence agent. It was showed that NO content in peroxisomes isolated from senescent pea leaves is less than that in young leaves [70]. NO emission is negatively correlated with senescence processes [35], [40]. Moreover, the NO-deficient mutant *nos1/noa1* exhibits an early-senescence phenotype and dark-induced senescence and chlorophyll loss can be rescued in *nos1/noa1* leaves by treatments with NO donors [43]. Interestingly, expressing a NO degrading enzyme in Arabidopsis led to early leaf senescence phenotypes with a massive up-regulation of *SENESCENCE-ASSOCIATED GENEs* (*SAGs*) [44]. In agreement with the above findings described for the role of NO in regulating Chl degradation and leaf senescence, our data support the model that decreases in NO levels occurring during leaf senescence under normal conditions relieve the repression of the enzymatic activities involved in Chl catabolic pathway at the transcriptional level, which is likely to be an adaptation for plants to accelerate Chl degradation in accordance with leaf senescence programming.

In addition to negatively regulating the expression levels of Chl catabolic pathway genes, our findings presented in this study have demonstrated that NO plays a positive role in maintaining stability of thylakoid membranes during dark-induced leaf senescence. This conclusion has been drawn based on several lines of evidence described in this study. Firstly, NO deficiency caused by NOS1/NOA1 mutations accelerates the stability loss of thylakoid membrane complexes during dark-induced leaf senescence by BN-PAGE. Secondly, western blotting data indicate that the abundance of thylakoid membrane proteins rapidly declined in the *nos1/noa1* mutant upon dark-treatment. Thirdly, in accordance with BN-PAGE and western blotting findings, NO deficiency leads to the collapsed ultrastructures of thylakoid membranes in chloroplasts of the darkened leaves. As the final stage of leaf development, leaf senescence undergoes a programmed cell death process, referred to as the senescence syndrome [65], [66]. Chl breakdown is a dramatically visualized sign of the leaf senescence syndrome [3], [71]. During senescence, the degeneration of chloroplasts and disruption in photosynthesis occurs first with deterioration and leakiness of the thylakoid membranes [63], [64], [66]. Importantly, our data have provided evidence to support that NO plays a dual role in regulating the process of leaf senescence as a negative regulator for Chl degradation pathway and as a positive regulator in maintaining the stability of thylakoid membranes. On the other hand, the analysis of the *pao1 nos1/noa1* double mutant has led us to reveal that the retained Chl levels caused by *PAO* mutations in the double mutant dose not improve the stability of thylakoid membranes with respect to the *nos1/noa1* mutant during dark-induced leaf senescence, indicating that NO is a determinant factor in regulation of the degradation of thylakoid membrane complexes during leaf senescence.

In conclusion, this study sheds light on the critical role of NO in regulating the stability of thylakoid membrane complexes in coordination with the degradation of the pigment apparatus during leaf senescence in the dark-induction course of yellowing of leaves. Considering that temporal progress of maturation and senescence goes hand in hand with a significant decrease of NO emission [35], [40], NO may play important roles in reprogramming the overall organization of senescing events such as Chl breakdown and the degeneration of chloroplasts with deterioration and leakiness of the thylakoid membranes. It is known that NO acts as a signaling molecule involving in a variety of biotic and abiotic stress responses in higher plants. Our studies also provide insight into better understanding the entire process of leaf senescence in response to environmental stresses.

## Materials and Methods

### Plant material and growth conditions

The Columbia (Col-0) ecotype of Arabidopsis (*Arabidopsis thaliana*) was used as the wild type. The T-DNA insertion mutant *nos1/noa1* was kindly provided by Dr. Nigel Crawford (University of California at San Diego, USA) and the mutant *pao1* (stock number: CS3733) was obtained from the Arabidopsis Biological Resource Centre (ABRC, Ohio State University, USA). The *pao1 nos1/noa1* double mutant was obtained by crossing and the putative double mutants were confirmed by performing PCR and sequence analyses. Plants were grown on peat soil under a long-day condition, 16 h of white light (80 µmol m^−2^ s^−1^) and 8 h in dark, with 60% relative air humidity at 21°C.

### Senescence induction

Fully extended rosette leaves were detached from 3-week-old plants and used for dark-induced leaf senescence. The detached leaves were incubated on Petri dishes containing three layers of filter papers soaked in 15 ml distilled water. Petri dishes were wrapped with double-layer aluminum foil, and kept in dark at 22°C.

### RT-PCR and real time RT-PCR

Total RNA was isolated using the Plant RNeasy kit (Qiagen). After DNA digestion with RQ1 DNase (Promega), first-strand cDNA was synthesized using the high efficient reverse transcription kit ReverTra Ace-α-® (TOYOBO). Quantitative real-time PCR was performed with SYBR Premix Ex TaqII (Takara) using a MyiQ5 single color Real-Time PCR Detection System (Bio-Rad). The comparative threshold cycle (Ct) method was used for determining relative transcript levels (iQ5 admin, Bio-Rad) using *ACTIN2* as an internal control. Specific primers to respective genes were as follows:

*ACT2* (forward, 5′- GCCATCCAAGCTGTTCTCTC-3′ and reverse, 5′- GCTCGTAGTCAACAGCAACAA-3′);

*SGR* (forward, 5′-GCAAGGATGGGCAAATAGG-3′ and reverse, 5′-CACCGCTTATGTGACAATGAAC-3′);

*PAO* (forward, 5′- ACGGCATGGTAAGAGTCAGC -3′ and reverse, 5′- AAACCAGCAAGAACCAGTCG -3′);

*RCCR* (forward, 5′- ATCGCCTCCAATCACAACTC-3′ and reverse, 5′- TTAGCACAAGCGACTTGGAA-3′);

*PPH* (forward, 5′- CAATCATGCTTGCTCCTGGTG-3′ and reverse, 5′- CTACCAATCCTGGACTCCTCC-3′);

*NYC1* (forward, 5′- ATGTGATGAGCAGACAGCACAGTG-3′ and reverse, 5′- CCTTAGCCAGGAAGTGACAATAGC-3′).

### PAO enzyme assays

The activities of PAO were measured using a coupled assay with RCCR according to previous reports, with minor modifications [11], [23], [72]. Briefly, leaf samples were processed (5 ml/g fresh weight) in a homogenization buffer containing 400 mM sorbitol, 25 mM Tricine-KOH, pH 8.0, 2 mM EDTA, 1 mM MgCl2, 0.1% bovine serum albumin (W/V), 5 mM polyethylene glycol 4000, and 0.5 mM dithiothreitol with chilled mortars and pestles. Next, the homogenized samples were filtrated through two layers of nylon cloth, and the resulting homogenate was centrifuged at 7,000 g for 4 min. The resulting green sediment was washed once with the washing medium (the homogenization medium with EDTA, MgCl2, and polyethylene glycol 4,000 omitted). After centrifugation at 8,000 *g* for 5 min, the resulting pellets were re-suspended in the washing medium (2 ml/g fresh weight leaf tissue). The re-suspended samples were further centrifuged at 14,000 *g* for 5 min. The resulting sediments were frozen in liquid nitrogen and stored at −80°C. According to the previous procedures, soluble stroma protein (S1 fraction) was used as the source of RCCR and solubilized membrane protein in osmotic buffer (Tris-MES, pH 8.0, 0.1% bovine serum albumin) with 1% Triton X-100 was used as the source of PAO, respectively.

To test the activities of PAO, Pheide *a* (30 µg), used as substrate, was mixed with the ferredoxin-reduced system containing 10 µg ferredoxin (Sigma), 1 mM NADPH (Sigma), 1 mM Glc-6-P (G6P), and 10 milliunits of G6P dehydrogenase. The final reaction mixture volume was 50 µl. After incubation in the dark at 25°C for 1 h, the reaction was terminated with methanol at a final concentration of 70%. The reaction mixture was centrifuged at 14,000 *g* for 10 min, and the supernatant was analyzed for pFCC-1 by reverse-phase HPLC with 0.1 M potassium phosphate (pH 7.0)/methanol (32.5%∶67.5%, V/V).

pFCC-1 was analyzed by HPLC with an Agilent 1100 HPLC ChemStation coupled to a fluorescence detector equipped with a ZorbaxSB C-18 column (4.6 mm×25 cm, 5-µm particle diameter). Samples (40 µl) were injected at a flow rate of 0.8 ml/min. Under these conditions, pFCC-1was eluted after 9 min. Fluorescence was recorded at 320 nm (excitation)/450 nm (emission). The amount of pFCC-1 was calculated according to integrated peak areas.

### Chlorophyll measurements

The fully extended rosette leaves detached from 3-week-old plants were used for dark-induced leaf senescence. Chlorophyll was extracted from the detached leaves after dark-induction. The chlorophyll in leaf samples was extracted with 80% (V/V) acetone. The chlorophyll contents were determined spectrophotometrically at 645 and 663 nm as described previously by [73].

### Chlorophyll fluorescence measurements

The maximum photochemical efficiency of PSII was determined from the ratio of variable (Fv) to maximum (Fm) fluorescence (Fv/Fm) with an LI-6400XT Portable Photosynthesis System (LI-COR Biosciences, Lincoln, Nebraska USA).

### Separation of thylakoid membranes and BN-PAGE

For the preparation of thylakoid membranes, leaves (0.5 g) were homogenized in 5 ml isolation buffer (400 mM sucrose, 2 mM MgCl_2_, 10 mM NaCl, and 50 mM HEPES, Ph 7.8) with chilled mortars and pestles. After filtrating through two layers of nylon cloth, samples were washed twice with the isolation buffer, and centrifuged at 5,000 *g* for 10 min. The resulting sediments were suspended in 100 µl re-suspension buffer (20% [w/v] glycerol and 25 mM BisTris-HCl, pH 7.0) and stored in −80°C for use. BN-PAGE was performed as described [74], [75].

### Western blot analysis

Immunodetection of thylakoid membrane proteins was performed using the indicated primary antibodies against thylakoid membrane proteins (Agrisera). Alkaline-phosphatase-conjugated goat anti-rabbit IgG (Chemicon) was used as a secondary antibody and reaction was revealed using an ECL kit (Amersham).

### Transmission electron microscopy

For transmission electron microscopy processing, the 5th leaves of the 21-d-old wild-type and mutant plants were collected. The leaf samples were fixed in 2.5% glutaraldehyde (v/v) for 48 h at 4°C. Thin sections were examined by a transmission electron microscope (H-7650, Hitachi) using a voltage of 80 kV.

### HPLC Analysis of Chlorophyll and Chlorophyll Catabolites

For pigment extraction, leave samples (about 0.1 g) were ground in a mortar with liquid nitrogen. Pigments were extracted with 10% (v/v) 0.2 M Tris-HCl, pH 8.0, in acetone. The extracts were incubated at −20°C for 2 h in the dark. After centrifugation at 14,000 *g*, 4°C for 20 min, 20 µl supernatants were analyzed on Agilent 1100 HPLC ChemStation as described [18], [76].

### Accession numbers for the sequence data and Arabidopsis seed stocks

Sequence data from this article can be found in the Arabidopsis Genome Initiative and GenBank/EMBL data libraries under accession numbers: *NOS1/NOA1* (At3g47450); *PAO* (At3g44880); *SGR* (At4g22920); *NYC1* (At4g13250); *PPH* (At5g13800); *RCCR* (At4g37000); *ACTIN2* (At3g18780). The ABRC seed stock numbers for the *nos1/noa1* and *pao1* mutants are CS6511 and CS3733, respectively.

## Supporting Information

Figure S1 Effects of SNP, an NO donor, on the transcript levels of chlorophyll breakdown pathway genes in the leaves of wild type and *nos1/noa1* mutant during dark-induced senescence. (A–E) qRT-PCR analysis of mRNA abundance of enzyme genes (*SGR*, *NYC1*, *PPH*, *PAO* and *RCCR*) involved in chlorophyll degradation in the leaves of wild type and *nos1/noa1* mutant treated with or without 250 µM SNP during dark-induced senescence. *ACTIN2* was used as the internal standard. Error bars indicate standard deviations of three technical replicates, and the results were consistent in three biological replicates.
(TIF)

Figure S2 Effects of cPTIO, an NO scavenger, on the transcript levels of chlorophyll breakdown pathway genes in wild type leaves during dark-induced senescence. (A–E) qRT-PCR analysis of mRNA abundance of enzyme genes (*SGR*, *NYC1*, *PPH*, *PAO* and *RCCR*) involved in chlorophyll degradation in wild type leaves treated with or without 500 µM cPTIO during dark-induced senescence. *ACTIN2* was used as the internal standard. Error bars indicate standard deviations of three technical replicates, and the results were consistent in three biological replicates.
(TIF)

Figure S3 Effects of cPTIO on abundance of thylakoid membrane protein complexes from wild type leaves incubated in dark. Blue native-PAGE analysis of thylakoid membrane protein complexes from the detached leaves of wild type after a 4-d-dark treatment in combination with or without 500 µM cPTIO.
(TIF)

Table S1 Analysis of PaO-RCCR activity in wild type and *nos1/noa1*. Detached leaves incubated in darkness for 4 days from both genotypes were used for crude extraction of PaO and RCCR. With all necessary cofactors, reaction mixtures were incubated at 25°C for 1 h and stopped by cold methanol. LU, Fluorescence unit.
(TIF)
