# Supplementary figures and images for: Nitric Oxide Deficiency Accelerates Chlorophyll Breakdown and Stability Loss of Thylakoid Membranes during Dark-Induced Leaf Senescence in Arabidopsis
Source: PLoS One. 2013 Feb 13;8(2):e56345. doi: 10.1371/journal.pone.0056345 (PMC3572010; doi:10.1371/journal.pone.0056345)

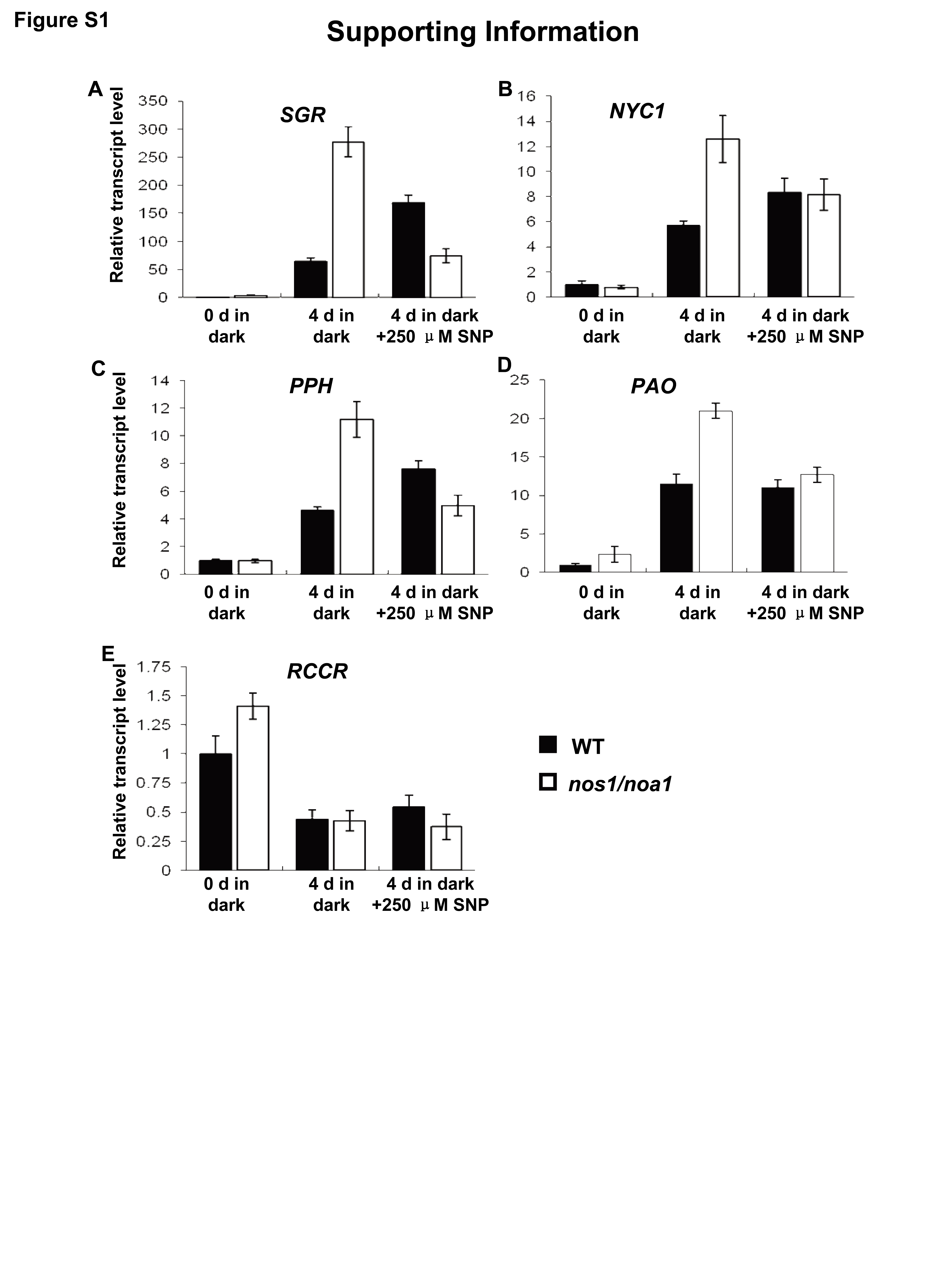

Supplement: Figure S1 — Effects of SNP, an NO donor, on the transcript levels of chlorophyll breakdown pathway genes in the leaves of wild type and nos1/noa1 mutant during dark-induced senescence. (A–E) qRT-PCR analysis of mRNA abundance of enzyme genes (SGR, NYC1, PPH, PAO and RCCR) involved in chlorophyll degradation in the leaves of wild type and nos1/noa1 mutant treated with or without 250 µM SNP during dark-induced senescence. ACTIN2 was used as the internal standard. Error bars indicate standard deviations of three technical replicates, and the results were consistent in three biological replicates. (TIF) [file pone.0056345.s001.tif]

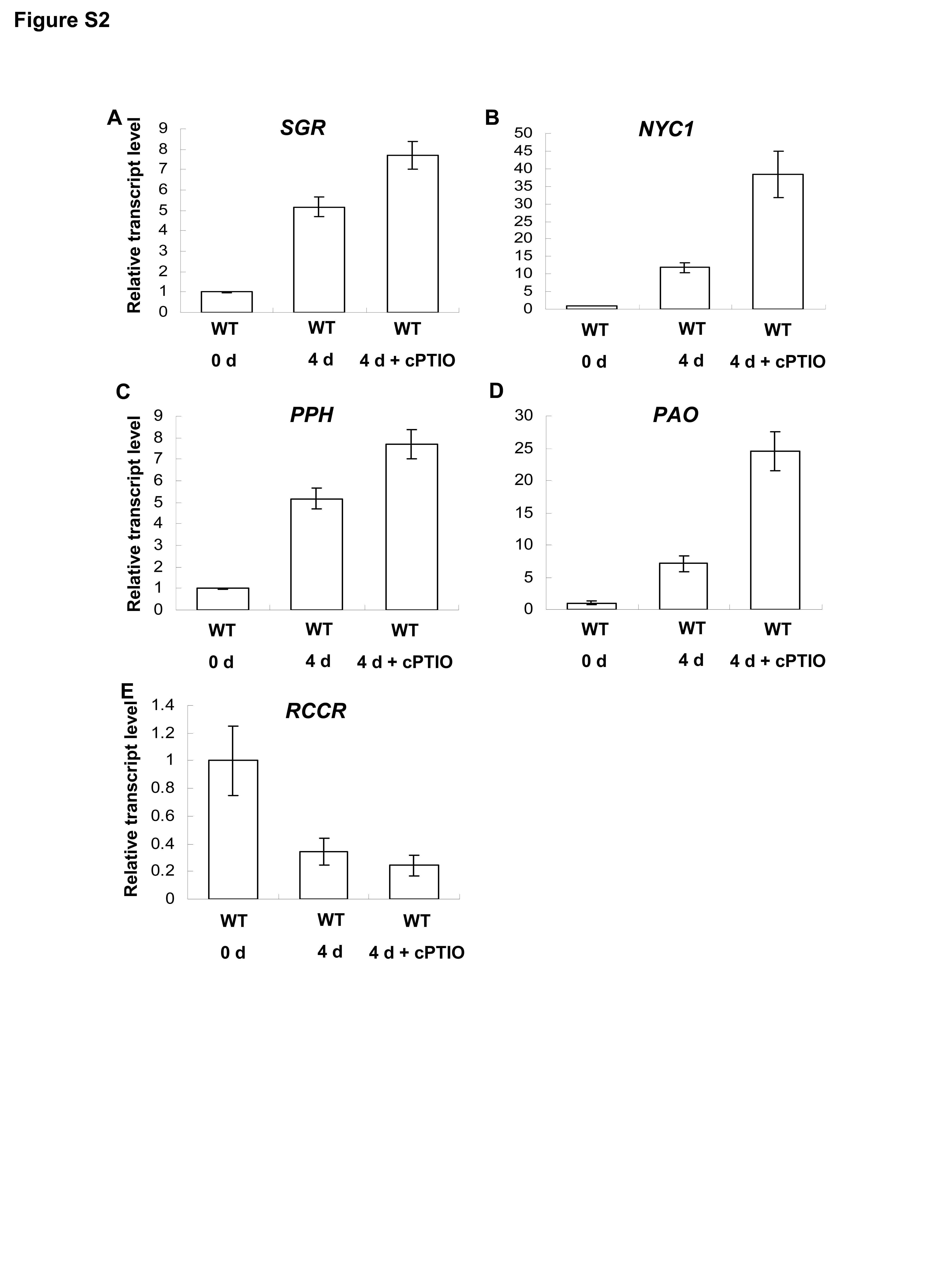

Supplement: Figure S2 — Effects of cPTIO, an NO scavenger, on the transcript levels of chlorophyll breakdown pathway genes in wild type leaves during dark-induced senescence. (A–E) qRT-PCR analysis of mRNA abundance of enzyme genes (SGR, NYC1, PPH, PAO and RCCR) involved in chlorophyll degradation in wild type leaves treated with or without 500 µM cPTIO during dark-induced senescence. ACTIN2 was used as the internal standard. Error bars indicate standard deviations of three technical replicates, and the results were consistent in three biological replicates. (TIF) [file pone.0056345.s002.tif]

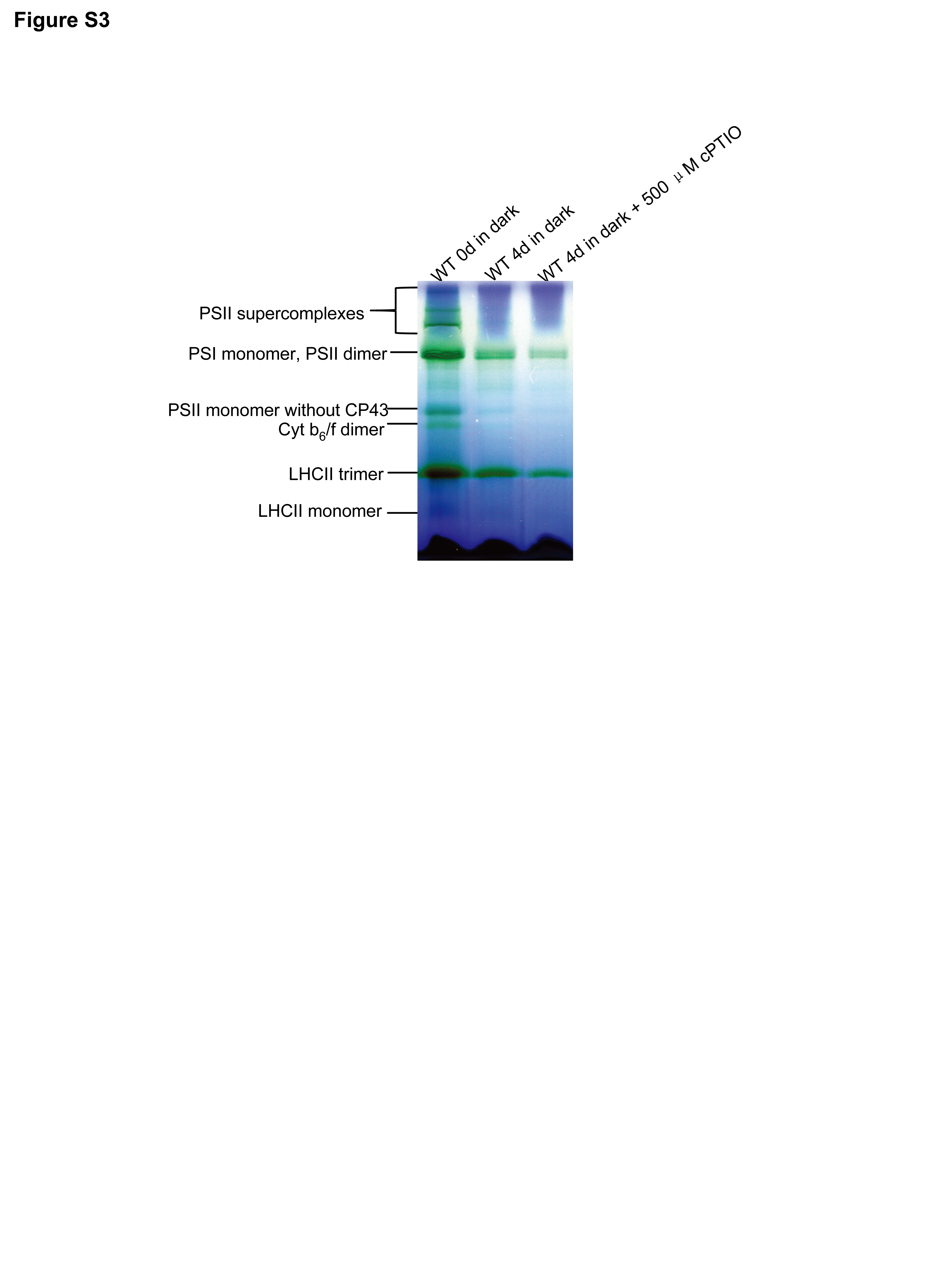

Supplement: Figure S3 — Effects of cPTIO on abundance of thylakoid membrane protein complexes from wild type leaves incubated in dark. Blue native-PAGE analysis of thylakoid membrane protein complexes from the detached leaves of wild type after a 4-d-dark treatment in combination with or without 500 µM cPTIO. (TIF) [file pone.0056345.s003.tif]

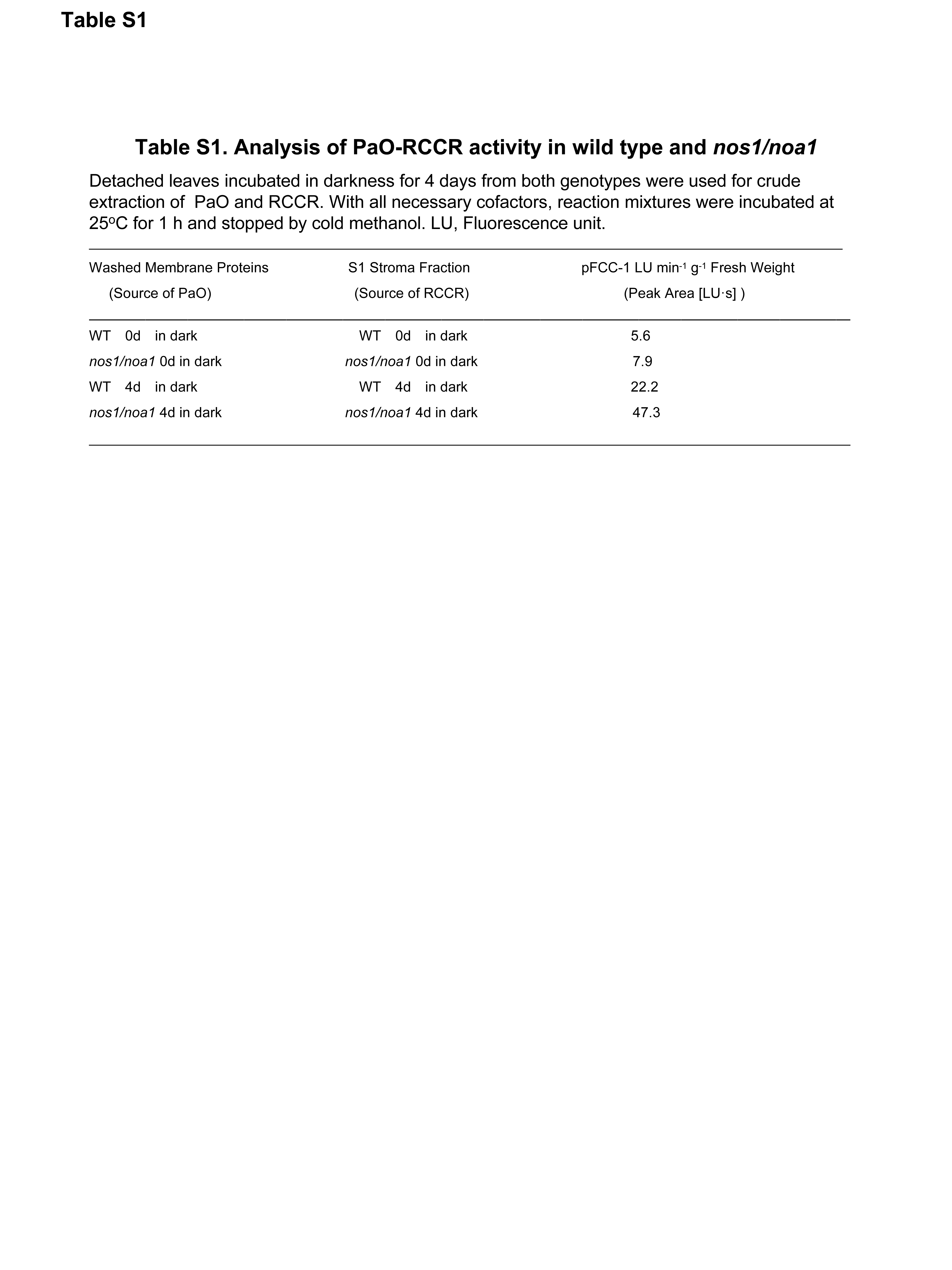

Supplement: Table S1 — Analysis of PaO-RCCR activity in wild type and nos1/noa1. Detached leaves incubated in darkness for 4 days from both genotypes were used for crude extraction of PaO and RCCR. With all necessary cofactors, reaction mixtures were incubated at 25°C for 1 h and stopped by cold methanol. LU, Fluorescence unit. (TIF) [file pone.0056345.s004.tif]
